# Supplementary figures and images for: Characterization of Molting Process during the Different Developmental Stages of the Diamondback Moth Plutella xylostella
Source: Insects. 2022 Mar 15;13(3):289. doi: 10.3390/insects13030289 (PMC8950211; doi:10.3390/insects13030289)

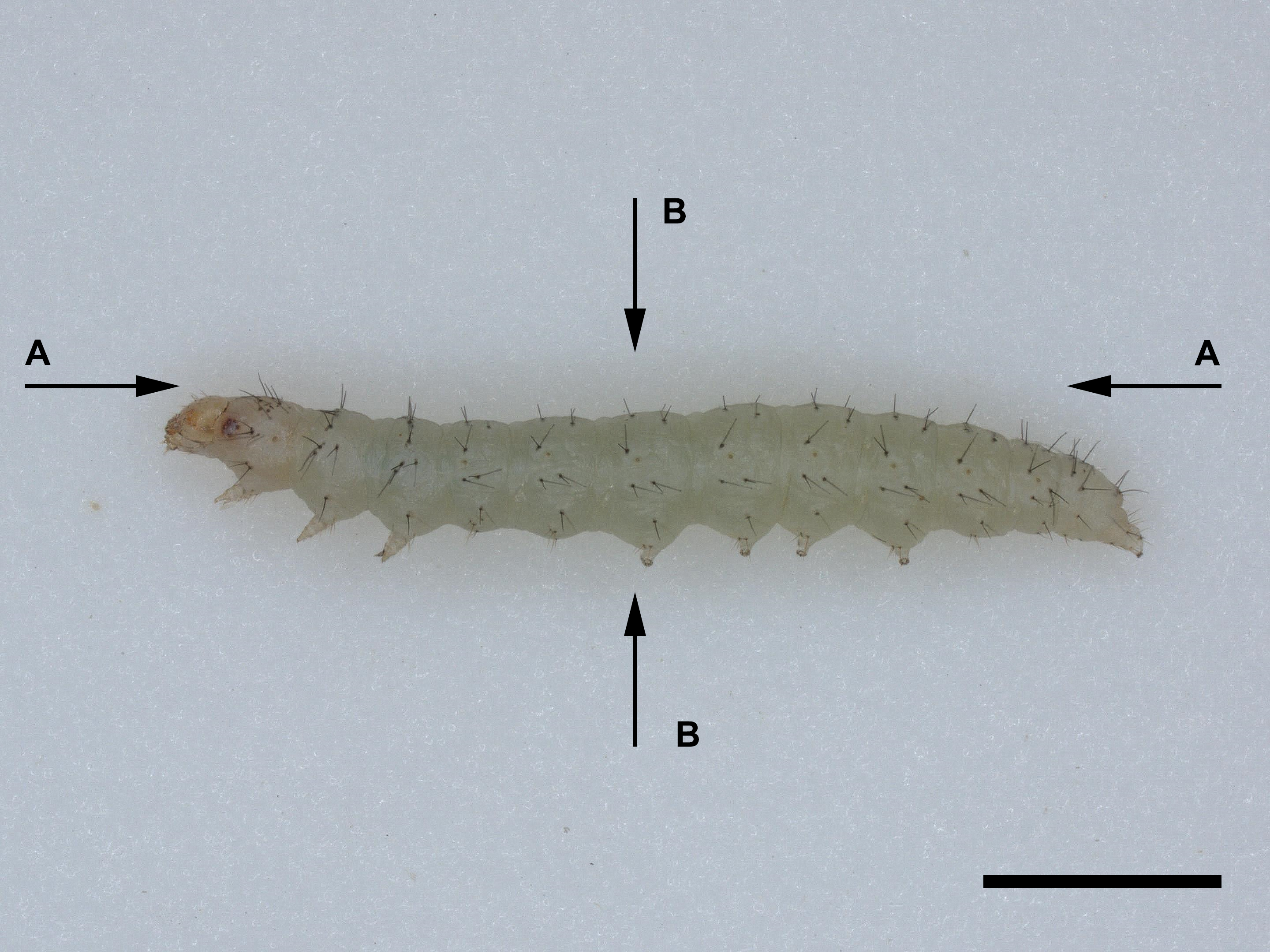

Supplement: Supplementary file 1 [file insects-13-00289-s001.zip › figureS1.tif]
